# *N*,*N*’-Bis(salicylidene)ethylenediamine (Salen) as an Active Compound for the Recovery of Ni(II), Cu(II), and Zn(II) Ions from Aqueous Solutions

**DOI:** 10.3390/membranes10040060

**Published:** 2020-04-02

**Authors:** Katarzyna Witt, Daria Bożejewicz, Małgorzata A. Kaczorowska

**Affiliations:** Faculty of Chemical Technology and Engineering, UTP University of Science and Technology, 3 Seminaryjna, PL 85326 Bydgoszcz, Poland; daria.bozejewicz@utp.edu.pl (D.B.); malgorzata.kaczorowska@utp.edu.pl (M.A.K.)

**Keywords:** *N*,*N*’-bis(salicylidene)ethylenediamine, salen, metal ions, nickel(II), copper(II), zinc(II), liquid–liquid extraction, polymer inclusion membranes, sorption

## Abstract

In this paper, three main methods of metal ion separation, i.e., liquid–liquid extraction, transport across polymer inclusion membranes (PIMs), and sorption/desorption, are described. In all of them, *N*,*N*’-bis(salicylidene)ethylenediamine (salen) was used as an active compound, i.e., as an extractant or as a carrier for the recovery of Ni(II), Cu(II), or Zn(II) ions from aqueous solutions. In each case, the recovery was performed on a model solution, which contained only a single metal ion. The obtained results were compared with the author’s previous results for the separation of metal ions using β-diketones, since both β-diketones and salen form the so-called Werner-type complexes. Electrospray ionization high-resolution mass spectrometry (ESI-HRMS) was also applied to confirm the ability of the carrier to form complexes with metal ions in a solution. Moreover, spectrophotometry was used to determine the stability constant of the obtained complexes.

## 1. Introduction

The structure of the compound molecule (ligand) has a huge influence on the process of forming its complexes as the ligand, being a chemical form directly connected with the central atom (metal ion), uses a free pair of electrons. Furthermore, this phenomenon is closely related to the possibility of using ligands as extractants or as carriers in separation processes such as liquid–liquid extraction or transport across polymer inclusion membranes, respectively. In both these processes, complex compounds of metal ions and ligands are formed. The stability of these complexes affects the efficiency of metal ion recovery from the aqueous solution. Ligands, which form very stable complexes, are successfully used as extractants in solvent extraction or precipitation processes. However, they cannot be used as carriers in PIMs, because binding between a ligand and a metal ion is so strong that the metal cannot be released to the receiving phase and, therefore, cannot be recovered.

Among many types of ligands, the most common are those that form the so-called Werner- type complexes [1]. This means that a metal ion is bound through one of three possible donor atoms of the ligand: nitrogen, oxygen, or sulfur. The ligands containing nitrogen and oxygen are imidazole [2] and trioctyl phosphine oxide (TOPO), respectively [3]. In turn, Cyanex 301 [4] and Cyanex 471X [5] have a sulfur atom in the structure. All of these are used in metal ion separation processes, e.g., in liquid–liquid extraction or transport across polymer inclusion membranes (PIMs).

Complexation reactions occur by binding one or more than one donor atoms. A ligand that has only one atom that coordinates directly to the central atom in a complex is called a monodentate [6]; in turn, a polydentate ligand (chelate) [7] is a structure that is attached to a central metal ion by bonds from two or more donor atoms.

The literature identifies many examples of polydentate ligands which form more stable complex compounds than monodentate ligands. For example, β-diketones are a group of compounds which bind cations by two oxygen atoms and form very stable chelates. LIX-54 is a well-known copper(II) extractant from the group of β-diketones [8].

**Figure 1:**
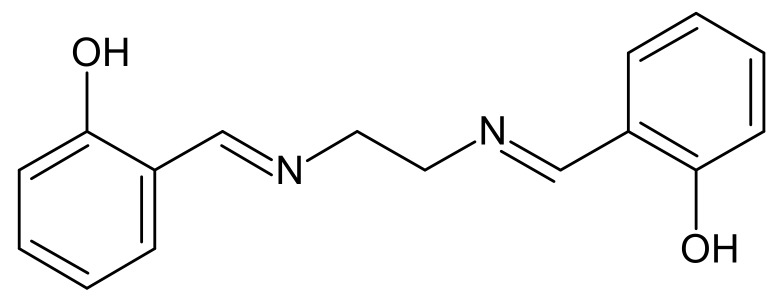
Structure of *N*,*N*’-bis(salicylidene)ethylenediamine (salen).

*N*,*N*’-Bis(salicylidene)ethylenediamine (salen) is also a polydentate ligand with oxygen and nitrogen donor atoms. Its structure is shown in Figure 1.

The literature states that salen easily forms complexes with various metal ions and that these complexes can have different applications. Earlier data on the complexes of salen were reported in 1966 by Gerloch et al. [9]. They described complexation reaction of ferric chloride with the Schiff base, i.e., salen in acetone solution. The obtained complex was monomeric in chloroform solution and non-conducting in nitromethane. Lewis et al. [10] reported the magnetic properties of some binuclear complexes of chromium(III) and iron(III). Furthermore, Chuguryan and Dzyubenko [11] used a spectrophotometric method for complexing neptunium with *N*,*N*’-bis(salicylidene)ethylenediamine in aqueous ethanol solutions in the range of pH = 6–10 and temperature of 25 ± 1 °C. They proved that, in the given conditions, only one mononuclear NpO_2_ (salen)^−^ complex could be prepared. The determined concentration stability constant was equal to 7.5 ± 0.035. They suggested using the studied compound for spectrophotometric analysis of neptunium in aqueous alcohol solutions. Vol’pin et al. [12] in turn considered the use of chelate cobalt(II) complex [Co(*N*,*N*’-bis(salicylidene)ethylenediamine)] as an effective catalyst for NADH (nicotinamide adenine dinucleotide) oxidation in methanol at room temperature. Nielson et al. [13] described the preparation of polymeric {TiO(*N*,*N*’-bis(salicylidene)ethylenediamine)}*_n_* from [Ti(OCHMe_2_)_2_(acac)_2_] and salen-H_2_ in methanol solution. Yang et al. [14] synthesized and characterized *N*,*N*-bis(salicylidene)ethylenediamine zinc(II) iodide (ZnLI_2_), and applied it as the electrolyte of a dye-sensitized solar cell. Huerta-Jose et al. [15] prepared and employed indium(III) complex of salen as a chemo-sensor for the recognition of HSO_4_^−^.

Finally, we found three papers in which salen was used for the removal of metal ions from solutions. Tadjorodi et al. [16] prepared BSEA–SBA-15 by covalently anchoring *N*,*N*’- bis(salicylidene)ethylenediamine (BSEA) on mesoporous silica (SBA-15) using a hydrothermal method. The authors suggested that BSEA–SBA-15 can be used in the future as an effective adsorbent for the removal of metal ions due to it having a porous structure and active functional groups. Dadfarnia et al. [17] synthesized silver ion-imprinted polymer, in the presence of an Ag(I)–salen complex, using 4-vinylpyridine as the functional monomer, ethylene glycol dimethacrylate as the crosslinker, and 2,2-azobis(isobutyronitrile) as the initiator. Then, the imprinted Ag(I) ions were completely removed by leaching. The polymer was employed as a selective sorbent for extraction and separation of the trace amounts of Ag(I) ions. Three years ago, Reffas et al. [18] studied the cloud point extraction for the purpose of separation of copper(II) from aqueous saline sulfate medium with salen as the chelating extractant and the polyethoxylated alcohol Tergitol 15-S-7 as the biodegradable non-ionic surfactant.

Based on the found data, due to its structure and insolubility in water, *N*,*N*’-bis(salicylidene)ethylenediamine can be used as an extractant or a carrier in both liquid–liquid extraction and transport across PIMs. That is why, in this paper, we considered the possibility of using salen in the above-mentioned processes. We also describe its use in sorption processes. The obtained complexes of salen and Ni(II), Cu(II), and Zn(II) were characterized using spectrophotometric and mass spectrometry methods.

## 2. Materials and Methods

### 2.1. Reagents

*N*,*N*’-Bis(salicylidene)ethylenediamine was purchased from Merck (Kenilworth, NJ, USA). Its selected properties are presented in Table 1.

The standard solutions of metal ions (Ni(II), Cu(II), and Zn(II)) were purchased from Sigma Aldrich (Poznan, Poland), while the aqueous solutions of metal ions (Ni(NO_3_)_2_, Cu(NO_3_)_2_, and Zn(NO_3_)_2_) were made from their analytical reagent grade salts (Avantor, Gliwice, Poland) in double-distilled water. The concentration of these solutions was standardized using the atomic absorption spectrometry method. The solution of analytical reagent grade nitric acid (Avantor, Gliwice, Poland) was standardized against anhydrous sodium carbonate. Ammonia was obtained from Avantor (Gliwice, Poland). Methanol, chloroform, and tetrahydrofuran (analytical reagent grade, Avantor, Gliwice, Poland) were used without further purification. Polyvinyl chloride (PVC) with an average molecular weight of 72,000 was obtained from ANWIL (Wloclawek, Poland). Bis(2-ethylhexyl)adipate (DAO) was purchased from Sigma-Aldrich (Poznan, Poland).

### 2.2. Stability Constants of Complexes of N,N’-Bis(salicylidene)ethylenediamine with Various Metal Ions

For calculation of stability constants of the complexes of salen with nickel(II), copper(II), and zinc(II) ions, a known spectrophotometric method was used [20]. The stock metal ions solutions were made from the appropriate standard solutions, and their concentration was equal to 0.1 g/L. The salen was dissolved in methanol, and the concentration of this stock solution was about 0.001 mol/L. Then, the spectrophotometric titration was carried out, and the absorption spectra of each prepared sample were recorded on a Cary 50 spectrophotometer (Varian, Melbourne, Victoria, Australia). The molar ratios of the components (salen with respect to Ni(II), Cu(II), or Zn(II)) were different in each sample. The spectra were recorded in the range of 200 to 500 nm.

### 2.3. Liquid–Liquid Extraction of Metal Ions from Model Solution Using N,N’-Bis(salicylidene)ethylenediamine

*N*,*N*’-Bis(salicylidene)ethylenediamine was used as an extractant during the performed liquid–liquid extraction of metal ions (Ni(II), Cu(II), or Zn(II)) from a model solution. The liquid–liquid extraction process was carried out in three variants with different salen–metal ion molar ratios (the concentration of metal ions in each case was constant):

I. 0.5:1 (3.6 × 10^−5^ mol of salen in organic phase with 7.2 × 10^−5^ mol of Ni(II), Cu(II), or Zn(II) ions in the aqueous phase);
II. 1:1 (7.2 × 10^−5^ mol of salen in organic phase with 7.2 × 10^−5^ mol of Ni(II), Cu(II), or Zn(II) ions in the aqueous phase);
III. 1.5:1 (10.8 × 10^−5^ mol of salen in organic phase with 7.2 × 10^−5^ mol of Ni(II), Cu(II), or Zn(II) ions in the aqueous phase).

All measurements were run at 25 °C, and a fixed ionic strength was maintained in the aqueous phase with 0.5 mol/L solution of potassium nitrate. The aqueous phase contained, except for various metal ions (Ni(II), Cu(II), or Zn(II)), ammonia to keep a suitable pH (12.5) during the extraction process. Chloroform was used as a solvent in the organic phase. The volume of the organic phase was always equal to the volume of the aqueous phase, which was 3.6 mL. The prepared samples were then shaken for one hour. The equilibrium was established after approximately 15 min. After checking if any changes in the phase volumes occurred, the phases were separated. The concentration of metal ions in the aqueous phase after liquid–liquid extraction was determined by atomic absorption spectrophotometry (AAS 240FS Spectrometer, Agilent, Santa Clara, CA, USA).

### 2.4. Polymer Inclusion Membranes with N,N’-Bis(salicylidene)ethylenediamine

#### 2.4.1. Preparation of Polymer Inclusion Membranes (PIMs)

The membranes were obtained by the casting method as described in our earlier paper [21]. All membranes were made of three components: polyvinyl chloride (PVC; as a polymer support), DAO (as a plasticizer), and *N*,*N*’-bis(salicylidene)ethylenediamine (as a metal ion carrier). Two types of compositions of the membranes were created: the first type with 20 wt.% salen and the second type with 40 wt.% salen. Smaller samples of the PIMs were cut out from the same membrane film to allow repeating the experiments. Obtained membranes were homogeneous and flexible.

#### 2.4.2. Sorption of the Metal Ions onto Polymer Inclusion Membranes

The sorption of the metal ions onto obtained polymer inclusion membranes was conducted according to the method described by Best et al. [22]. Membranes were immersed in a beaker containing 50 mL of solution, which was made in the same way as the feed phase described in Section 2.4.4. The solution was shaken using a magnetic stirrer at a speed of 50 rpm. Samples of the solution were taken at regular time intervals and diluted using nitric acid; then, the concentration of metal ion was determined using the AAS method.

#### 2.4.3. Desorption of the Metal Ions from Polymer Inclusion Membranes

The desorption of the metal ions from the surface of PIMs was performed by rinsing the membrane after the sorption process (Section 2.4.2) with distilled water; after drying, it was immersed in 30 mL of 0.05 mol/L aqueous nitric acid. Reduced volumes of solutions were used to concentrate the initial solutions from Section 2.4.2. Further operations were the same as in Section 2.4.2.

#### 2.4.4. Transport Studies across Polymer Inclusion Membranes

The transport experiments were carried out in a permeation module cell, which was also presented in the authors’ earlier paper [21]. As a feed phase, an aqueous solution containing 0.02 mol/L of metal ions (Ni(II), Cu(II), or Zn(II)) and ammonia was used. Ammonia was added to keep a suitable pH (12.5) during the process. As a receiving phase, the 0.05 mol/L aqueous solution of nitric acid was used.

### 2.5. Analysis of Complexes of N,N’-Bis(salicylidene)ethylenediamine with Various Metal Ions by Mass Spectrometry

The high-resolution mass spectrometry (HRMS) experiments were performed using a Q-Exactive Orbitrap mass spectrometer (Thermo Fisher Scientific, Bremen, Germany) equipped with a TriVersa NanoMate robotic nanoflow ESI ion source (Advion BioSciences ltd., Ithaca, NY, USA). Samples of the separated organic phases after liquid–liquid extraction described in detail in Section 2.3, in variant III, were diluted (1:1) in methanol (Avator, Gliwice, Poland). MS data were acquired in a positive ion mode within the *m*/*z* range of 50–750 at the resolution of 140,000 (*m*/*z* 200). Obtained mass spectra were processed in Thermo Xcalibur software (ver. 4.1.31.9).

## 3. Results and Discussion

### 3.1. Complexation Properties of N,N’-Bis(salicylidene)ethylenediamine

The dissociation constant of salen was found in the ChemicalBook database. Its value is equal to pK_a_ = 12.59 ± 0.50. According to the Brønsted–Lowry theory, the value of dissociation constant K_a_ of the ligand determines its basicity and tendency to dissociate a proton. At this pH, the proton can be easily replaced with other cations in a complexation reaction. This means that salen can be used as an extractant or as a carrier for the removal of metal ions from strong alkalic solutions, e.g., from wastewater from concrete companies [23], electroplating companies [24], or breweries [25].

The increase in value of K_a_ of the ligand causes an increase in the stability constant of its complexes [26].

The stability constants of the complexes of salen with Ni(II), Cu(II), and Zn(II) ions consisted of one mole of ligand and one mole of metal ion (type 1:1). A summary is presented in Table 2.

The obtained log β_1_ showed similar values. Small variations can be seen. The close proximity of the studied metals in the periodic table may explain this fact. The stability of a complex correlates with the size of ionic radius of the involved metal ion [27,28]. The obtained values of stability constants were inversely proportional to the values of ionic radii of their metal ions: Ni^2+^ (r Ni^2+^ = 72 pm) < Cu^2+^ (r Cu^2+^ = 69 pm) > Zn^2+^ (r Zn^2+^ = 74 pm). The highest stability constant was obtained for the complex of salen with copper(II) ions. The values of stability constants of salen complexes with nickel(II) and zinc(II) ions were lower than those for copper(II) complexes. 

A similar situation occurred in the case of β-diketone. For example, the dissociation constant of acetylacetone in a water–methanol solution is equal to 9.65 [29], and the stability constants of its complexes with nickel(II), copper(II), and zinc(II) are 4.96, 8.24, and 5.76 [29], respectively. The dissociation constant of acetylacetone is lower than the pKa of salen, but the changes in stability constants for both compounds with particular metal ions are similar, except for the zinc(II) complex.

### 3.2. Separation Processes

In this paper, we describe the following separation processes: liquid–liquid extraction, transport across polymer inclusion membranes, and sorption/desorption. In all of them, *N*,*N*’-bis(salicylidene)ethylenediamine was used as an active compound (extractant or carrier) for the recovery of the investigated metal ions from solution. In each process, the recovery was performed on a model solution which contained only a single metal ion (e.g., Ni(II)).

In the sections below, the parameters of all mentioned processes are quantified and discussed.

#### 3.2.1. Liquid–Liquid Extraction

**Figure 2:**
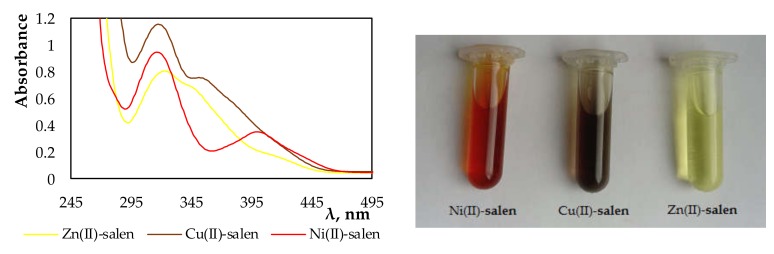
Absorption spectra and photography of organic phases after extraction using salen (variant III).

The spectra and photographs below (Figure 2) clearly show that, during the extraction, the complexes of the investigated metal ions with salen used as an extractant were formed. The complexes of nickel(II), copper(II), and zinc(II) ions with salen in chloroform solutions had specific colors of red, purple, and yellow, respectively.

In order to analyze the process of extraction of metal ions from the simple model solution, further calculations were made. At the beginning, the distribution ratios (*D_M_*) of metal ions for each investigated system were calculated using Equation (1).

(1) $$D_{M}=\frac{C_{M\left(org\right)}}{C_{M\left(aq\right)}}=\frac{C_{M}^{0}-C_{M}}{C_{M}}$$

where *C*^0^*_M_* and *C_M_* denote the analytical metal ion concentrations in the aqueous phase before and after attaining a partition equilibrium (mol/L), respectively.

Then, the percentage of metal ion extraction (%E) was calculated using Equation (2) for each of the investigated systems.

(2) $$\%E=\frac{D_{M}\cdot 100\%}{D_{M}+\frac{V_{aq}}{V_{org}}}$$

where *V_aq_* and *V_org_* are the volumes of aqueous and organic phases, respectively (*V_aq_*/*V_org_* = 3.6 mL/3.6 mL = 1).

Table 3 shows the values of *D_M_* and %E, which were calculated for each of the three experiment variants with different salen–metal ion molar ratios.

The received results show that the values of D_M_ and %E increased along with the increasing concentration of salen in the samples. The highest percentage of extraction (99.79%) was obtained for copper ions, whereas %E for zinc and nickel ions extracted in the same conditions was 87.68% and 60.98%, respectively. The obtained results proved that *N*,*N*’-bis(salicylidene)ethylenediamine can be successfully used as an extractant for metal ion recovery, especially for the recovery of copper(II) ions. For comparison, the values of %E obtained in our previous experiment [30], regarding the extraction of copper ions from ammonia solution using 3-allyl-acetylacetone, 3-butyl-acetylacetone, and unsubstituted acetylacetone as extractants, were lower and equal to 85%, 80%, and 72%, respectively.

#### 3.2.2. Sorption

The analysis of the metal ion sorption process onto the membranes with 20 wt.% and 40 wt.% of salen was carried out using Equation (3).

(3) $$q_{t}=\frac{C_{}^{i}-C_{}^{t}}{m}\cdot V$$

where *q_t_* denotes the sorption capacity (mg/g), V is the volume of the solution (L), m is the mass of the sorbent (g), and *C^i^* and *C^t^* are analytical metal ion concentrations in the solution at the beginning and after an appropriate time of sorption process (mol/L), respectively.

**Figure 3:**
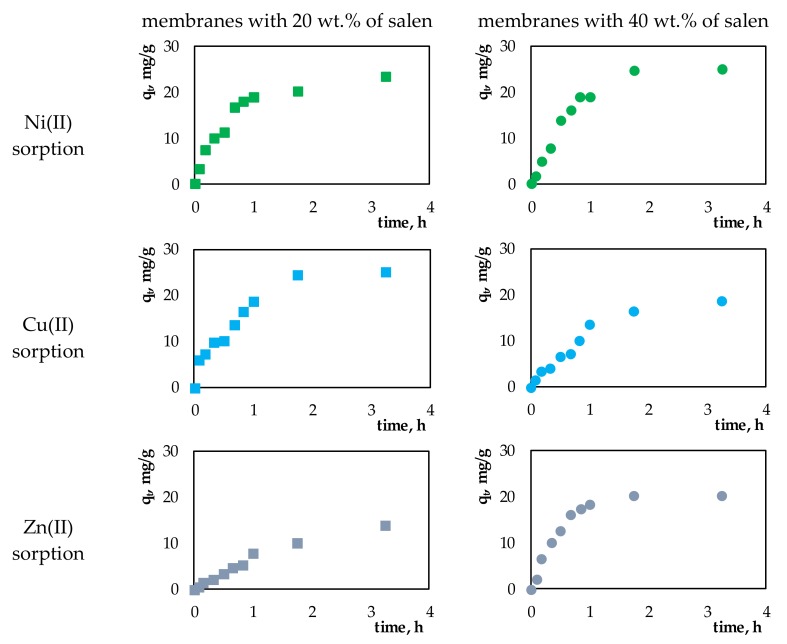
The changes in sorption capacity of the membranes with 20 wt.% or 40 wt.% salen during the sorption processes.

Here, *q_t_* describes the amount of metal ions adsorbed on the surface of the membrane over a specific period of time. Figure 3 presents plots of *q_t_* vs. time for sorption processes of the investigated Ni(II), Cu(II), or Zn(II) ions onto the membranes containing 20 wt.% or 40 wt.% salen.

In all of investigated sorption processes, during the first hour, a rapid increase in sorption capacity was observed (*q_t_*). This fact can be related to the large number of available active places in relation to the amount of sorbed metal ions. After that time, the adsorption of metal ions onto the surface of membranes slowed down, eventually reaching equilibrium.

After 24 h of sorption, the percentage of metal ion removal from the solutions (%R_s_) was also determined (Equation (4)).

(4) $$\%R_{s}=\frac{C_{}^{i}-C_{}^{t}}{C_{}^{t}}\cdot 100\%$$

where *C^i^* and *C^t^* denote analytical metal ion concentrations in the solution at the beginning and after an appropriate time of sorption process (mol/L), respectively.

**Figure 4:**
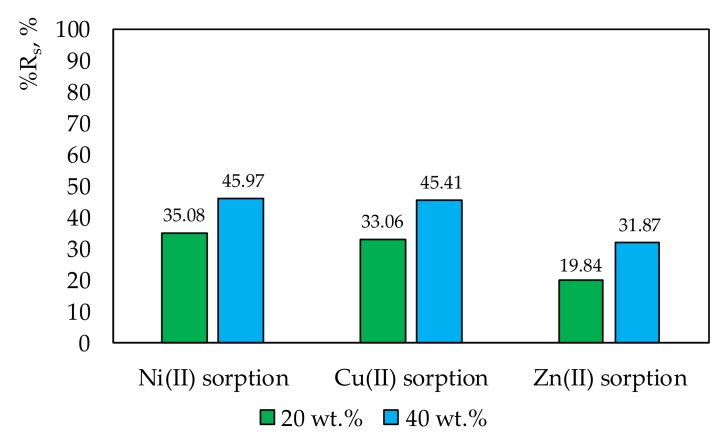
The percentage of metal ion removal from the solutions after 24 h of sorption processes onto membranes with 20 wt.% or 40 wt.% salen.

The %Rs reached the values presented in Figure 4.

Figure 4 shows that the effectiveness of sorption correlated with the increasing amount of ligand in the membrane. A 20% increase in the amount of salen in the membrane increased metal ion sorption on its surface by about 10-fold. The sorption effectiveness of copper or nickel ions was almost the same on the membrane with 20 wt.% and 40 wt.% salen.

**Figure 5:**
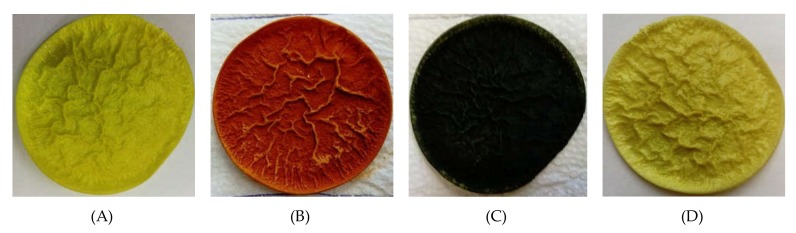
Photography of polymer inclusion membranes (PIMs) with 40 wt.% salen: (**A**) before sorption; (**B**) after sorption from solution with Cu(II) ions; (**C**) after sorption from solution with Ni(II) ions; (**D**) after sorption from solution with Zn(II) ions.

Figure 5 shows the visible result of sorption on membranes. The colors of deposits on the membranes were the same as those obtained in organic phases of the liquid–liquid extraction. This confirms the formation of complexes of the investigated metal ions with salen present in the structure of the membrane.

In our previous paper [31], we considered the possibility of removing Cu(II), Zn(II), and Pb(II) ions via sorption on PVC-based composite materials with various contents of acetylacetone. The reduction in ion concentration ranged from 8% to 91%, from 10% to 85%, and from 6% to 50% for Cu(II), Zn(II), and Pb(II) ions, respectively, depending on the composite composition. The best results were obtained for the material containing 30 wt.% acetylacetone, as well as porophor, which increased the active surface of the material.

#### 3.2.3. Desorption

The desorption of Ni(II), Cu(II), and Zn(II) ions from the surface of membranes with salen, used during the sorption processes, to the solution of nitric acid at a concentration of 0.05 mol/L was also performed. The results presented in Table 4 were calculated as a percentage of the sum of desorbed metal ions from the sum of previously adsorbed metal ions.

Almost full desorption in nitric acid was achieved only for zinc(II) ions in the case of the membrane with 20 wt.% salen, probably because that membrane bound the smallest amount of ions during sorption. It is observed that, in the case of membranes where large amounts of metal cations were absorbed, the desorption process was complicated (the average percentage of desorption was equal to approximately 30%).

#### 3.2.4. Transport across Polymer Inclusion Membrane

In order to describe the efficiency of metal removal from the feed phase, the recovery factor (%RF) was calculated (Equation (5)).

(5) $$\%RF=\frac{C^{0}-C_{r}}{C^{0}}\cdot 100\%,$$

where *C^0^* is the initial concentration of metal ions in the feed phase (mol/L), and *C_r_* is the concentration of metal ions in the receiving phase after time (mol/L). 

The results obtained after 24 h of metal ion transport across PIMs doped with salen in relation to various amounts of the carrier are shown in Table 5.

The results presented in Table 5 show that the recovery of the investigated metal ions using PIMs with salen is minimal in the applied conditions. 

The process of metal ion transport across a membrane is described by a linear kinetic equation (Equation (6)).

(6) $$\ln\left(\frac{C}{C^{0}}\right)=-kt,$$

where *C^0^* is the initial concentration of metal ions in the donor phase (mol/L), *C_r_* is the concentration of metal ions in the donor phase after time t (mol/L), *k* is the rate constant (h^−1^), and *t* is the time of transport process (h).

The following values also describe metal ion transport across PIMs:

• Initial flux (*J*_0_):

(7) $$J_{0}=\frac{V}{A}kC^{0},$$

where *J*_0_ (µmol/m^2^s) denotes the initial flux at *t* = 0, *V* is the volume of the receiving phase (m^3^), and *A* is the interface of membrane (m^2^);

• Permeability coefficient (*P*) [m/s]:

(8) $$P=-\frac{V}{A}k.$$

The results of the calculation of the above parameters are shown in Table 6.

The obtained results show that permeability coefficients and initial fluxes for metal ion transport across the studied membranes were low (slightly higher in the case of the membrane with 40 wt.% salen). This fact explains the low values of recovery factors (%RF), which were described above. The highest recovery factors were obtained for nickel(II) ions (5.87% and 10.63% during the transport across PIMs with 20 and 40 wt.% salen, respectively), and nickel(II) ions were also transported with the highest initial fluxes by both membranes. This shows that the compositions of the received membranes were not suitable for the transport of the investigated metal ions and must be changed.

### 3.3. Mass Spectrometry

**Figure 6:**
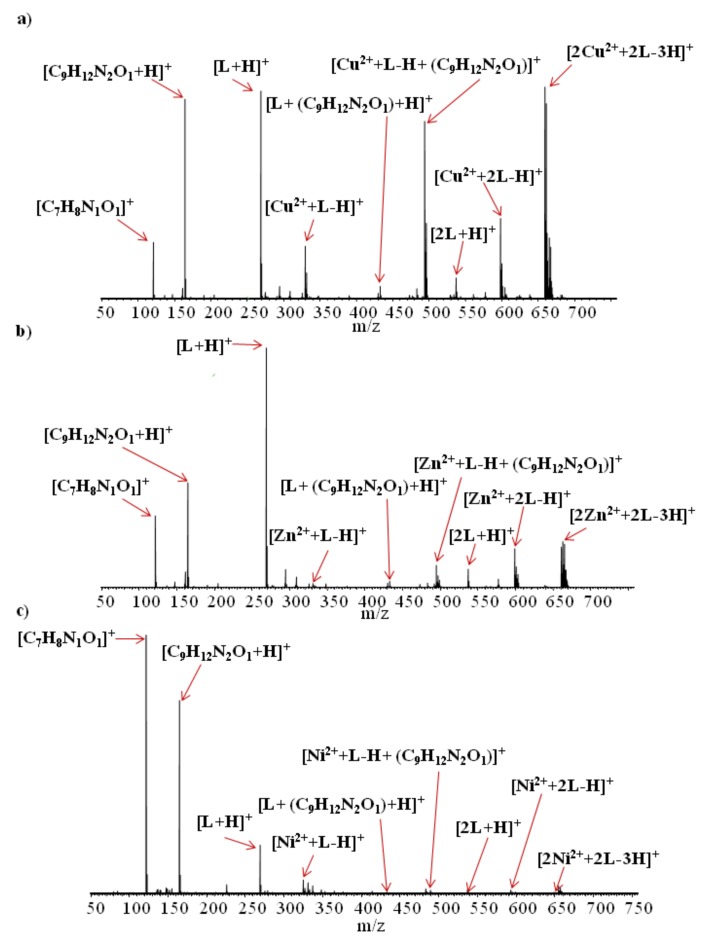
ESI-HRMS spectra recorded for the samples of the separated organic phases after liquid–liquid extraction, containing *N*,*N*’-bis(salicylidene)ethylenediamine (L) and metal ions (**a**) Cu^2+^, (**b**) Zn^2+^, and (**c**) Ni^2+^. Unassigned minor signals correspond to ions not relevant in this study (e.g., formed by solvent molecules).

Electrospray ionization high-resolution mass spectrometry experiments (ESI-HRMS) were performed for the separated organic phases obtained after liquid–liquid extraction of metal ions (Cu^2+^, Zn^2+^, and Ni^2+^) from model solutions, using *N*,*N*’-bis(salicylidene)ethylenediamine (this procedure was described in detail in Section 2.3, in variant III). ESI-HRMS spectra of the analyzed samples are shown in Figure 6a–c, while the ESI-HRMS data of the main compounds found are presented in Table 7.

The results of ESI-HRMS experiments show that, for all of the analyzed samples in the solutions after extraction, similar complexes containing metal ions and *N*,*N*’-bis(salicylidene)ethylenediamine (L) molecule(s), were formed (i.e., [Cu^2+^ + L − H]^+^, [Zn^2+^ + L − H]^+^, [Ni^2+^ + L − H]^+^, [Cu^2+^ + 2L − H]^+^, [Zn^2+^ + 2L − H]^+^, [Ni^2+^ + 2L − H]^+^, [2Cu^2+^ + 2L − 3H]^+^, [2Zn^2+^ + 2L − 3H]^+^, [2Ni^2+^ + 2L − 3H]^+^). However, the intensity of signals corresponding to complexes of the same type formed by different metal ions was not the same. For example, signals which can be assigned to [2Cu^2+^ + 2L − 3H]^+^ ions were dominant (Figure 6a), and those corresponding to the [2Zn^2+^ + 2L − 3H]^+^ were much less intense (Figure 6b), whereas the signals corresponding to [2Ni^2+^ + 2L − 3H]^+^ (Figure 6c) ions were minor. The same reduction in intensity was observed for all signals corresponding to similar complexes differing only by metal ions. This allows for the conclusion that, although the properties of metal ions do not affect the types of complexes formed, they have a large impact on the quantity of generated complex ions. These findings are consistent with the liquid–liquid extraction results, where the highest percentage of extraction was obtained for copper and the lowest was obtained for nickel ions.

Electrospray ionization of all of the examined solutions also led to the formation of singly charged ions related to *N*,*N*’-bis(salicylidene)ethylenediamine, such as [L + H]^+^, [2L + H]^+^, [C_7_H_8_N_1_O_1_]^+^, and [C_9_H_12_N_2_O_1_ + H]^+^. Ions [C_7_H_8_N_1_O_1_]^+^ and [C_9_H_12_N_2_O_1_ + H]^+^ were probably formed as a result of partial L molecule decomposition in the solution, during electrospray ionization process or during compound storage. The ESI-HRMS spectra show that fragments of L could also form complexes with both metal ions (e.g., [Cu^2+^ + (L − H) + (C_9_H_12_N_2_O_1_)]^+^, [Zn^2+^ + (L − H) + (C_9_H_12_N_2_O_1_)]^+^, [Ni^2+^ + (L − H) + (C_9_H_12_N_2_O_1_)]^+^) and whole L molecules (e.g., [L + (C_9_H_12_N_2_O_1_) + H]^+^).

Based on the results of the performed ESI-HRMS experiments, it is possible to conclude that, in the analyzed solutions, various types of complexes of copper(II), zinc(II), and nickel(II) ions with *N*,*N*’-bis(salicylidene)ethylenediamine molecule(s) were formed. Given the high mass accuracy of the HRMS mass spectrometry, there can be no question as to the elemental composition or charge of the ions generated. However, it should be emphasized that the ESI-HRMS method does not allow the detection of neutral complexes that can also be formed in the examined solutions.

## 4. Conclusions

The value of the dissociation constant of *N*,*N*’-bis(salicylidene)ethylenediamine (salen) is equal to pKa = 12.59 ± 0.50, which determines the basicity of the compound and tendency to dissociate a proton. That proton can be easily replaced with other cations in a complexation reaction. The obtained stability constants of complexes of salen with Ni(II), Cu(II), and Zn(II) ions were similar since the stability correlates with the size of the ionic radius of the involved metal ion.

The results obtained in liquid–liquid extraction show that salen is a very effective extractant, especially for removing copper(II) ions from aqueous solutions, but its efficiency depends on its concentration in the system. The obtained polymer inclusion membranes with salen can be used as sorbents for the recovery of metal ions from solutions, but they cannot be successfully used as metal ion carriers in transport across those membranes. Electrospray ionization high-resolution mass spectrometry (ESI-HRMS) was successfully used to confirm the ability of *N*,*N*’-bis(salicylidene)ethylenediamine to form complexes with Cu^2+^, Zn^2+^, and Ni^2+^ ions in solution. The results of the performed ESI HRMS experiments provide information about the elemental composition of formed complexes.

## Figures and Tables

**Table 1:** Selected properties of *N*,*N*’-bis(salicylidene)ethylenediamine (salen) [19].

Form	Crystal
Color	Yellow
Assay	98%
Melting point	127–128 °C
Boiling point	411.48 °C
Solubility in water	Insoluble
Solubility in chloroform	0.1 g/mL

**Table 2:** Values of stability constants (logβ_1_) of Ni(II), Cu(II), and Zn(II) complexes with *N*,*N*’-bis(salicylidene)ethylenediamine in an aqueous methanol solution at 25 °C.

Ligand	log β_1_		
	Ni(II)	Cu(II)	Zn(II)
*N*,*N*’-bis(salicylidene)ethylenediamine	5.438	5.452	5.426

The given values carry ±0.001 tolerance.

**Table 3:** The distribution ratios (*D_M_*) and the percentages of metal ion extraction (%E) of the investigated systems in ammonia solutions.

Variant of Experiment/salen:M	*D_M_*			%E (%)		
	Ni(II)	Cu(II)	Zn(II)	Ni(II)	Cu(II)	Zn(II)
I/0.5:1	0.40	2.04	0.72	28.54	67.14	41.89
II/1:1	0.89	72.79	4.41	47.02	98.64	81.52
III/1.5:1	1.56	486.00	7.12	60.98	99.79	87.68

The given values carry ±0.01 tolerance.

**Table 4:** The percentage of the sum of desorbed metal ions after 24-h desorption of Ni(II), Cu(II), and Zn(II) ions from the surface of membranes with salen in a 0.05 mol/L solution of nitric acid.

	Membranes with 20 wt.% Salen	Membranes with 40 wt.% Salen
Ni(II)	36.59	20.69
Cu(II)	39.67	74.19
Zn(II)	94.51	39.22

The given values carry ±0.01 tolerance.

**Table 5:** Recovery factors (%RF) for the transport of investigated metal ions across polymer inclusion membranes (PIMs) with salen as a carrier.

	%RF (%)	
	Membranes with 20 wt.% Salen	Membranes with 40 wt.% Salen
Ni(II)	5.87	10.63
Cu(II)	3.26	9.35
Zn(II)	3.69	9.09

The given values carry ±0.01 tolerance.

**Table 6:** Permeability coefficients and initial fluxes for competitive transport of Ni(II), Cu(II), and Zn(II) ions across PIMs doped with salen.

		*P* 10^6^ (m/s)	*J*_0_ 10^5^ (µmol/m^2^s)
Membranes with 20 wt.% salen	Ni(II)	6.43	12.86
	Zn(II)	4.00	7.99
	Cu(II)	3.52	7.05
Membranes with 40 wt.% salen	Ni(II)	19.17	38.33
	Zn(II)	14.53	29.07
	Cu(II)	14.33	28.65

The given values carry ±0.01 tolerance.

**Table 7:** ESI-HRMS data of the main compounds found in the samples of the separated organic phases after liquid–liquid extraction (variant III described in Section 3.2) diluted (1:1) in methanol. L stands for *N*,*N*’-bis(salicylidene)ethylenediamine (C_16_H_16_N_2_O_2_).

**Cu^2+^ and L**		
***m*/*z*_meas_**	***m*/*z*_calc_**	**Assignment**
122.0599 165.1019 269.1279 330.0414 433.2224 494.1361 537.2487 598.1622 659.0757	122.0606 165.1028 269.1290 330.0429 433.2239 494.1379 537.2501 598.1641 659.0780	[C_7_H_8_N_1_O_1_]^+^ [C_9_H_12_N_2_O_1_ + H]^+^ [L + H]^+^, (C_16_H_17_N_2_O_2_)^+^ [Cu^2+^ + L − H]^+^, (CuC_16_H_15_N_2_O_2_)^+^ [L + (C_9_H_12_N_2_O_1_) + H]^+^, (C_25_H_29_N_4_O_3_)^+^ [Cu^2+^ + L − H + (C_9_H_12_N_2_O_1_)]^+^, (CuC_25_H_27_N_4_O_3_)^+^ [2L + H]^+^, (C_32_H_33_N_4_O_4_)^+^ [Cu^2+^ + 2L − H]^+^ (CuC_32_H_31_N_4_O_4_)^+^ [2Cu^2+^ + 2L − 3H]^+^ (Cu_2_C_32_H_29_N_4_O_4_)^+^
**Zn^2+^ and L**		
***m*/*z*_meas_**	***m*/*z*_calc_**	**Assignment**
122.0600 165.1019 269.1278 331.0408 433.2226 495.1361 537.2487 599.1620 661.0720	122.0606 165.1028 269.1290 331.0424 433.2239 495.1374 537.2501 599.1636 661.0771	[C_7_H_8_N_1_O_1_]^+^ [C_9_H_12_N_2_O_1_ + H]^+^ [L + H]^+^, (C_16_H_17_N_2_O_2_)^+^ [Zn^2+^ + L − H]^+^, (ZnC_16_H_15_N_2_O_2_)^+^ [L + (C_9_H_12_N_2_O_1_)+H]^+^, (C_25_H_29_N_4_O_3_)^+^ [Zn^2+^ + L – H + (C_9_H_12_N_2_O_1_)]^+^, (ZnC_25_H_27_N_4_O_3_)^+^ [2L + H]^+^, (C_32_H_33_N_4_O_4_)^+^ [Zn^2+^ + 2L − H]^+^ (ZnC_32_H_31_N_4_O_4_)^+^ [2Zn^2+^ + 2L − 3H]^+^ (Zn_2_C_32_H_29_N_4_O_4_)^+^
**Ni^2+^ and L**		
***m*/*z*_meas_**	***m*/*z*_calc_**	**Assignment**
122.0599 165.1019 269.1276 325.0472 433.2228 489.1452 537.2483 593.1677 649.0877	122.0606 165.1028 269.1290 325.0486 433.2239 489.1436 537.2501 593.1699 649.0895	[C_7_H_8_N_1_O_1_]^+^ [C_9_H_12_N_2_O_1_ + H]^+^ [L + H]^+^, (C_16_H_17_N_2_O_2_)^+^ [Ni^2+^ + L − H]^+^, (NiC_16_H_15_N_2_O_2_)^+^ [L + (C_9_H_12_N_2_O_1_) + H]^+^, (C_25_H_29_N_4_O_3_)^+^ [Ni^2+^ + L – H + (C_9_H_12_N_2_O_1_)]^+^, (NiC_25_H_27_N_4_O_3_)^+^[2L + H]^+^, (C_32_H_33_N_4_O_4_)^+^ [Ni^2+^ + 2L − H]^+^ (NiC_32_H_31_N_4_O_4_)^+^ [2Ni^2+^ + 2L − 3H]^+^ (Ni_2_C_32_H_29_N_4_O_4_)^+^
